# AMELX Gene Association with Dental Caries in Iranian Adults

**DOI:** 10.22088/IJMCM.BUMS.8.4.294

**Published:** 2019

**Authors:** Fatemeh Koohpeima, Maryam Derakhshan, Mohammad Javad Mokhtari

**Affiliations:** 1 *Department of Operative Dentistry, Biomaterial Research Center, School of Dentistry, Shiraz University of Medical Sciences, Shiraz, Iran.*; 2 *Young Researchers and Elite Club, Department of Biology, Zarghan Branch, Islamic Azad University, Zarghan, Iran.*

**Keywords:** Dental caries, DMFT, AMELX, rs946252, tetra-primer ARMS-PCR

## Abstract

Dental decay is a disease that is greatly affected by environmental components, but recently there have been an increasing number of documents supporting a genetic factor in the development of caries. The purpose of this study was to examine the association between dental caries and single-nucleotide polymorphisms in the *AMELX* gene. This research was carried out on 360 individuals of both sexes, who were referred to the dental school at the Shiraz University of Medical Sciences. In this research, individuals aged 20–65 years were divided into two groups: controls (decayed, missed, or filled teeth (DMFT) ≤ 5; n = 180) and cases (DMFT ≥ 14; n = 180). The tetra-primer ARMS-PCR technique was performed for genotyping the DNA extracted from blood cells. Analysis of the *AMELX* rs946252 polymorphism showed that the T allele of rs946252 was a significant protective factor against dental caries in Iranian adults (T vs. C: OR = 0.70, 95% CI: 0.49–0.98, P = 0.04). We demonstrated the significant differences in the genotype frequencies under two genetic models: overdominant (TC vs. TT + CC: OR 0.35, 95% CI 0.19–0.64, P = 0.0006) and recessive (CC vs. TC + TT: OR 2.57, 95% CI 1.39–4.76, P = 0.002). Our results show that the SNPs of the *AMELX *gene may be related with susceptibility to dental caries in Iranian adults.

Dental caries, which is also known as cavities or tooth decay, is determined as a process that causes the loss of the mineral component of the enamel and dentin structure (1). Despite more than many years of knowledge accumulated on the pathogenesis of caries, this disease is still an important oral health problem in most developed countries (2). This disease can produce pain, infection, tooth loss, and different other severe diseases. Many people are sensitive to caries progression throughout their lifetimes (2, 3). It is widely believed that dental decay is mostly affected by environmental factors, such as the diet and pH of the oral cavity, which is related to bacterial growth. However, there also is strong evidence suggesting a genetic component in caries susceptibility (3). The existence of certain types of disturbances in tooth formation can lead to the altered calcification or development of the enamel matrix. These enamel defects can influence a single tooth or the entire dentition (4).

Information on the genetic contribution to variation in the liability to caries has been indicated in investigations showing heritability estimates of about 50 percent (5, 6). There are different genes involved in the formation of the human dental enamel, i.e., *ENAM*, *AMELX*, *MMP-20*, *KLK-4*, and, more recently, *LX3*, *FAM83H*, *WDR72*, and *SLC4A4* (7). Amelogenin (AMEL) is the main matrix of proteins that has an important role in the formation of enamel. It represents about ninety percent of the organic content. *AMELX* and *AMELY * genes are responsible for encoding this protein, and are located on the X and Y chromosomes, respectively (8). *AMELY* is not under a strong functional constraint and is expressed at a low level. The *AMELX* gene contains seven exons and six introns. During enamel development, the AMELX protein is responsible for biominer-alization. Different studies indicate that genetic variation can lead to mineral losses of dental structures, which contribute to alterations of the enamel, bacterial attachment, and biofilm deposition (9). A new investigation of AMEL sequences has shown that different locations of this protein have developed differently over two hundred million years (9). Several studies have shown that exon 6 of *AMELX* gene is variable, while the N- and C-terminal regions are well-conserved (10).

Single-nucleotide polymorphisms (SNPs) are very important markers in different studies that link phenotypic changes to DNA-sequence variations. Studies in this field are expected to promote the analysis of human system physiology, and explain the molecular basis of diseases. The purpose of this study was to determine whether dental caries in Iranian adults is related to SNPs in the *AMELX* gene.

## Materials and methods

**Study subjects**

This research was performed on 360 individuals of both sexes (180 females and 180 males) referred to dental school at the Shiraz University of Medical Sciences. The ages of the examined persons were between 20 and 65 years. The individuals voluntarily agreed to participate in this venture as a part of a large prospective research project and completed the written informed consent form. The study was approved by the Ethics Committee of the Medical University of Shiraz, Iran. The examination of the dental and oral status was performed with the WHO criteria for all individuals by an expert dentist. In the same way, dental caries was explored by using a flat mirror and explorer, artificial light, and conventional dental chairs. In addition, the radiographic exam was performed as a part of the diagnosis of caries for all individuals by taking right and left bitewing radiographs; for teeth with dentin lesion with possible pulp lesion, periapical radiographs were used as a complementary diagnostic method. Dental status was scored by the number of decayed, missed, or filled teeth (DMFT). According to the DMFT, the individuals of this study were divided into two groups: cases and controls. Cases were defined as individuals diagnosed with high caries (DMFT ≥ 14; n=180), while controls were defined as individuals with lower caries and no history of caries (DMFT ≤ 5; n = 180) (11). Inclusion criteria consisted of patients aged between 20 and 65 years; it included men or women, and all subjects had a similar oral hygiene habits including fluoride exposure, access to preventive dental care, tooth brushing frequency, sugar intake and socioeco-nomic status. The individuals voluntarily signed an informed consent form that was approved by the Ethics Committee of the Medical University of Shiraz, Iran. Exclusion criteria consisted of a history of alcohol intake and smoking, existence of any systemic disease, history of any tooth-loss from traumatic injury, severe periodontal disease or orthodontic treatment, pregnant women, and existence of severe mental disorder. 

**SNP genotyping**

The salting out method was used to isolate DNA from the whole blood samples, and DNA purity was measured spectrophotometry using the Eppendorf Biophotometer (Germany). The *AMELX* genomic sequence was obtained from NCBI site (http://www.ncbi.nlm.nih.gov). The polymorphisms were detected and primers were designed for T-ARMS-PCR, which is a simple and rapid technique for the recognition of SNPs (12). The location of SNPs and T-ARMS-PCR primers are shown in Table 1. PCR reactions were performed in a total volume of 20 μl which contained 0.5 μM of each primer, 250 μM dNTPs, 1 U Taq DNA polymerase, 1.5 mM MgCl_2_, and 50 ng genomic DNA. The cycling conditions of PCR included initial denaturation at 95 °C for 5 min, followed by 30 cycles for rs946252 at 95°C for 30 s, annealing at 60 °C for 30 s, and extension at 72 °C for 40 s, with a final extension of 72 °C for 10 min. The PCR products were verified on 2% agarose gels. To ensure genotyping quality, approximately 20% of the random samples were sequenced and showed no genotyping error.

**Statistical analysis**

Hardy–Weinberg equilibrium (HWE) was assessed using the chi-square test. The difference of sex between the case and control groups was determined by the chi-square test method. This test also was used for the comparison of genotypes and allele frequencies in the two groups. We also used unconditional logistic regression analysis to test the relation between each SNP and case/control status under different genetic models, such as dominant, codominant, recessive, and overdominant. The results were considered statistically signiﬁcant when P was less than 0.05. All analyses were done using the version 19.0 of SPSS software.

## Results

The study group consisted of 180 high caries cases (90 females—50 percent and 90 males—50 percent), aged 20 to 65-years-old (the average age was 25.84 ± 7.91 years), and the mean of DMFT was 14.76. The controls were composed of 180 individuals (90 females—50 percent and 90 males—50 percent) who were with low caries, aged 20 to 65 years old (26.21 ± 9.45 years), and the mean of DMFT was 4.95. There was no statistical difference in age between the cases and controls (P> 0.05). Ten individuals were caries-free.

The genotypes and alleles frequencies of the *AMELX* rs946252 gene polymorphism in cases and controls are shown in Table 2. The control samples were not at HWE. The results confirmed strong supporting evidence for the association between the dental caries and rs946252 polymorphism in females. We found that the T allele of rs946252 was a significant protective factor against dental caries in Iranian adults (T vs. C: OR = 0.70, 95% CI: 0.49–0.98, P=0.04). We demonstrated signifi-cant differences in the genotype frequencies under the two genetic models: overdominant (TC vs. TT + CC: OR = 0.35, 95% CI 0.19–0.64, P = 0.0006) and recessive (CC vs. TC + TT: OR = 2.57, 95% CI 1.39–4.76, P = 0.002). No significant differences were observed between genotype frequencies and caries experience risk in males (OR = 1.27, 95% CI: 0.69–2.34, P = 0.43). The DMFT index was analyzed according to gender in both case and control groups. This index was not significant in case and control groups (Table 3).

**Table 1:** The primers used for detection of rs946252 polymorphism in *AMELX *gene

**Gene polymorphism**	**Product size**	**Primers**	**Sequence (5' to 3')**
*AMELX *rs946252	Outer primers: 331 bp	FO	TTTATGGAGCATTCATTACATCCATGTT
		RO	ATCAGAATTTTTGGTAGCGGCATTTGAG
	C allele: 160	FI	TTTCACAAACTTGGACATAAAAATCTTCC
	T allele: 229	RI	TGTTTTAAACCCTAATTTCACCAACTAGGA

**Table 2:** Distribution of genotypes and allele frequencies of the rs946252 polymorphism

**Gender**	**Genetic Model**	**rs946252**	**Cases**	**Controls**	**OR (%95CI)**	**P**
		**Polymorphism**	**(%)**	**(%)**		
Female		Genotype				
		TT	12 (13.33)	9 (10.00)	1	
	Co dominant	TC	32 (35.55)	55 (61.11)	0.43 (0.16-1.14)	0.08
		CC	46 (51.11)	26 (28.88)	1.32 (0.49-3.56)	0.57
	Dominant	TT	12 (13.33)	9 (10.00)	1	
		TC+CC	78 (86.66)	81 (90.00)	0.72 (0.28-1.80)	0.48
	Recessive	TC+TT	44 (48.88)	64 (71.11)	1	
		CC	46 (51.11)	26 (28.88)	2.57 (1.39-4.76)	0.002
	Over dominant	TT+CC	58 (64.44)	35 (38.88)	1	
		TC	32 (35.55)	55 (61.11)	0.35 (0.19-0.64)	0.0006
Male		Genotype				
		T	30 (33.33)	35 (38.88)	1	
		C	60 (66.66)	55 (61.11)	1.27 (0.69-2.34)	0.43
Female and Male		Allele				
		T	86 (31.85)	108 (40.00)	1	
		C	184 (68.14)	162 (60.00)	1.42 (1.02-2.03)	0.04

**Table 3:** Gender distribution for DMFT index

		**Male**	**Female**
**Mean **	Control	4.92±0.47	4.97±0.14
**P-value **	P>0.05		
**Mean **	Case	14.85±5.43	14.66±4.50
**P-value **	P>0.05		

## Discussion

Dental caries and periodontal disease are the two most common oral diseases in humans. They can cause systemic as well as local complications, such as pregnancy complications, preterm delivery, low birth weight, and early childhood caries (13, 14). Tooth decay is a multifactorial and complex disease process that causes suffering to a large percentage of the population. Host, microorganism, time, and substrates are important factors that produce caries (15, 16). It has been demonstrated that different genes such as those influencing the composition of saliva or genes taking part in formation of dentin and enamel, carbohydrate metabolism and influencing immune responses, have significant role in etiology of dental caries (17-19). Enamel development is a result of the expression of different complex genes that are needed to manage the complicated process of mineralization. AMEL is the most extracellular matrix protein of developing enamel (20). Many recent investigations focused on the role of genes in tooth formation. They showed that the genes responsible for enamel formation may have an important role in caries susceptibility. It was demonstrated that variation in these genes may cause different enamel defects by making structural changes in enamel (21, 22). However in our recent study, we find no relationship between *ENAM* gene polymorphism and caries experience (23). Specific isoforms of AMEL produced by alternative splicing serve definite signaling functions within the tooth structure development (24). Hence, different phenotypical enamel malformations may be created by mutations in *AMELX* (25). 

In this investigation, we studied whether the SNP in *AMELX* is associated with dental caries in female and male Iranian adults using the tetra-primer ARMS-PCR. This technique is an accurate, reliable, and simple method for genotyping SNPs. This method includes a PCR reaction in a vial with two pairs of primers, and is followed by electrophoresis on agarose gel. The rs946252 SNP is located in the intron region of the *AMELX*. Genetic alterations could lead to the abnormal functioning of cellular proteins and this change could result in the malformation of the enamel prisms that increase the susceptibility to dental decay. The data of the present study suggests that association can be detected between *AMELX* and caries experience. We found that the T allele of rs946252 was a significant protective factor against dental caries in Iranian adults. There was a significant association between rs946252 T > C and dental caries susceptibility under recessive (CC vs. TC+TT) and overdominant (TC vs. TT+CC) genetic models. Yet, no significant association was found between rs946252 and dental caries susceptibility under the dominant (TC+CC vs. TT) genetic model. 

It seems that the phenotype of the dental enamel is associated with *AMELX* mutation (26). Furthermore, mutations in *AMELX* led to the genetic and phenotypical diversity of X-linked amelogenesis imperfecta (27). As many previous investigations demonstrated the significance of the *AMELX* gene in enamel formation, certain authors proposed an association between *AMELX* gene polymorphism and dental caries. For example, in a Guatemalan–Mayan population, AMEL was related with a high-caries susceptibility (21) and in Turkish children, the genes that are responsible for enamel formation associated with high-caries experience (22). Although, sequencing *AMELX* in seventy Turkish children did not suggest this gene as an important candidate for caries experience (28). Another study in Turkish adults has shown no significant association between dental caries and *AMELX* (+522) polymorphism (11). Kang et al. have demonstrated that TT genotypes in *AMELX* (rs5933871; rs5934997) can lead to increased caries susceptibility (29). In addition, Gasse et al. in a large cohort study, demonstrated that the sequencing of all the coding regions of the *AMELX* in high caries children did not show an important polymorphism related to increased caries experience. In this SNP, (rs946252), the alleles frequency was C/T (0.63/0.37) (30). In the present study, the alleles frequency was C/T (0.68/0.32) in the case group and (0.60/0.40) in the control group. These results differ slightly from the previous reports produced by many studies. 

In conclusion, our findings showed that rs946252 polymorphism in the *AMELX *gene is associated with dental caries susceptibility, although, the development of dental decay necessitates a complex interaction between environmental and genetic variables. Supplem-entary studies are needed to demonstrate the potential function of this SNP and to validate these findings in other ethnic populations. Our study has some limitations such as small sample size, which may be inadequate for assessing statistical interaction. Considering the limited sample size, these results need to be replicated in other and larger populations. On the other hand, we tested only one SNP that may be considered as another limitation. 
